# Condensation Flow Heat Transfer Characteristics of Stainless Steel and Copper Enhanced Tubes

**DOI:** 10.3390/ma16051962

**Published:** 2023-02-27

**Authors:** Xu Wang, David John Kukulka, Wei Li, Weiyu Tang, Tianwen Li

**Affiliations:** 1 College of Energy and Transportation Engineering, Inner Mongolia Agricultural University, 306 Zhaowuda Road, Hohhot 010018, China; 2 Department of Mechanical Engineering Technology, State University of New York College at Buffalo, 1300 Elmwood Avenue, Buffalo, NY 14222, USA; 3 Department of Energy Engineering, Zhejiang University, 38 Zheda Road, Hangzhou 310027, China; 4 ZJU-Hangzhou Global Scientific and Technological Innovation Center, Hangzhou 311200, China

**Keywords:** condensation heat transfer, enhanced tube, heat transfer coefficient, pressure drop

## Abstract

In order to study the heat transfer of R410A in extreme environments, the properties of several stainless steel and copper-enhanced tubes were evaluated using R410A as the working fluid, and the results were compared with those of smooth tubes. Tubes evaluated include: smooth, herringbone (EHT-HB) and helix (EHT-HX) microgroove, herringbone/dimple (EHT-HB/D); herringbone/hydrophobic (EHT-HB/HY); and composite enhancement 1EHT (three-dimensional). Experimental conditions include a saturation temperature of 318.15K with a saturation pressure of 2733.5 kPa; a mass velocity in the range between 50 and 400 kg/(m^2^·s); and an inlet quality controlled at 0.8 and an outlet quality of 0.2. Results indicate that the EHT-HB/D tube produces the best overall condensation heat transfer characteristics (high heat transfer performance and low frictional pressure drop). Using the performance factor (PF) to compare tubes for the range of conditions considered, the *PF* of the EHT-HB tube is greater than one, the *PF* of the EHT-HB/HY tube is slightly greater than one, and the *PF* of the EHT-HX tube is less than one. In general, as the mass flow rate increases, *PF* initially decreases and then increases. Previously reported smooth tube performance models that have been modified (for use with the EHT-HB/D tube) can predict the performance for 100% of the data points to within ±20%. Furthermore, it was determined that the thermal conductivity of the tube (when comparing stainless steel and copper) will have some effect on the tube-side thermal hydraulic performance. For smooth tubes, the heat transfer coefficients (HTC) of copper and stainless steel tubes are similar (with copper tube values being slightly higher). For enhanced tubes, performance trends are different; the HTC of the copper tube is larger than the SS tube.

## 1. Introduction

Passive enhanced heat transfer technology (i.e., enhanced surfaces) can significantly increase heat transfer with only a small pressure drop increase. Therefore, this is an important technology to consider in the field of heating, ventilation, and air conditioning (HVAC). Enhanced micro-fin tubes have been a topic of study for many researchers.

Micro-fin shape/form (including the various geometric structure parameters of the fin) are of interest; the effect of these parameters on heat transfer performance has been the topic of many studies. Kim et al. [1] examined the effect of micro-fin tube diameter on the boiling heat transfer characteristics of horizontal flow. As tube diameter increases, the HTC of micro-fin tubes is significantly higher than that of smooth tubes. Wellsandt and Vamling [2] found that the HTC of Y-shaped, micro-fin tubes is slightly larger than that of traditional spiral micro-fin tubes; however, there is a higher pressure drop in these tubes (when compared with smooth tubes). Wu et al. [3] experimentally studied the flow boiling of HTC in five different enhanced micro-fin heat exchange tubes. Experimental results [for mass flow rates less than 400 kg/(m^2^·s)] show that micro-fin tubes with fin heights of 0.15 mm and fin apex angles of 25 and 30 degrees produce the best heat transfer performance. After studying the effect of the internal spiral angle on micro-fin tubes, Yang et al. [4] proposed an improved diagram that describes the internal flow pattern for horizontal micro-fin tubes; the transition curve to annular flow was determined to occur earlier in the micro-fin tube than what is found in the smooth tubes. Rollmann et al. [5] developed a novel pressure drop correlation (based on R407c and R410A pressure drop data) for micro-fin tubes.

With the recent development of enhanced three-dimensional tubes, studies of evaporation and condensation have become important topics to study. Li et al. [6] evaluated the condensation heat transfer characteristics of micro-fin tubes and compared them with smooth tubes. Kukulka et al. [7,8] experimentally evaluated changes in two-phase heat transfer for various enhanced tubes; they presented HTC and frictional pressure drop data for several enhanced three-dimensional tubes. Li et al. [9] compared the HTC of several stainless steel (SS) enhanced-surface tubes. Zhang et al. [10] carried out a similar analysis for a different set of conditions. Gu et al. [11] experimentally studied condensation heat transfer for moist air flows in enhanced tubes. Tang et al. [12] compared the condensation flow patterns found in a three-dimensional enhanced tube and detailed the transition conditions. Zhao et al. [13], Ali et al. [14], and Ji et al. [15] studied the effect that tube material thermal conductivity had on heat transfer of the fluid; Zhao et al. [13] studied carbon steel, copper, nickel, aluminum, and brass tube materials; Ali et al. [14] studied copper, brass, and bronze tube materials; and Ji et al. [15] discussed the relationship between fin thermal conductivity and fin efficiency.

Li et al. [16] studied the condensation of copper and SS-enhanced three-dimensional tubes using an R134a working medium. The HTC of the enhanced tube was 1.4~1.6 times higher than that of the smooth tube. A new correlation was presented for the enhanced tube that predicted the deviation of the condensation HTC in the tube to be ± 15%. Zheng et al. [17] carried out an experimental study on the evaporation characteristics of 410a in an enhanced three-dimensional SS tube. The largest evaporation HTC values were found in the EHT-HB/HY tube. Stratified wavy flow was found at the inlet of all tube types; the outlet flow pattern of the EHT-HB/HY and EHT-HB/D tubes was stratified wavy flow, while the outlet pattern of the EHT-HB tube was semi-annular flow, and for the EHT-HX tube it was annular flow. Kukulka et al. [18] presented the condensation heat transfer ratio (enhanced tube HTC/smooth tube HTC) of the enhanced tubes to be 1.15~2.05. Additionally, they found that the increase in heat transfer for enhanced tubes made of small tube diameters and/or high thermal conductivity materials was even larger. Some of the data used for comparison in this study came from Zheng et al. [17] and Kukulka et al. [18] and can be regarded as the continuation of those studies [16,17,18].

For the design, development, and assessment of high-performance heat transfer systems, it is important to study the heat transfer data of three-dimensional tubes. Additionally, the performance of enhanced tubes may or may not be better than that of smooth tubes. In order to determine how well enhanced tubes conduct heat and how material type may affect condensation heat transfer, an experimental study is necessary; additionally, in order to utilize enhancement results in industrial designs, an engineering model must be developed and validated. Smooth, herringbone (EHT-HB), and spiral (EHT-HX) microgrooves; herringbone/dimple (EHT-HB/D) herringbone/hydrophobic (EHT-HB/HY); and three-dimensional (composite dimple) 1EHT tubes are evaluated in the current study; their condensation heat transfer performance was analyzed and evaluated.

## 2. Experimental Details

**Figure 1:**
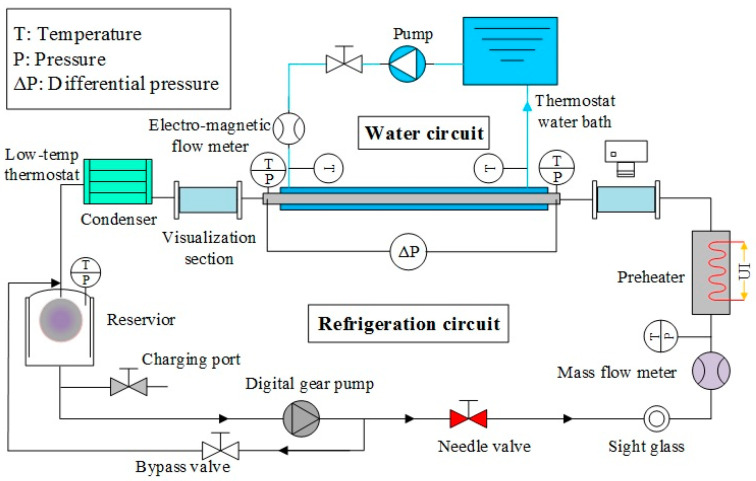
Experimental setup used in this study.

**Figure 2:**
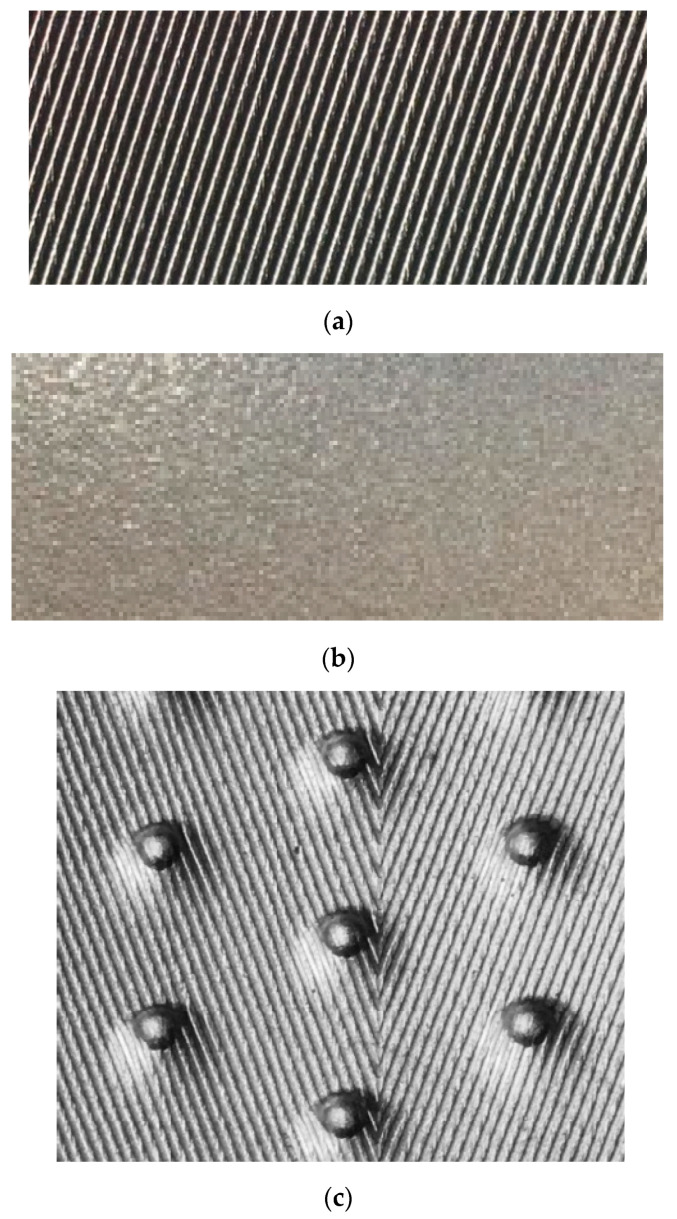

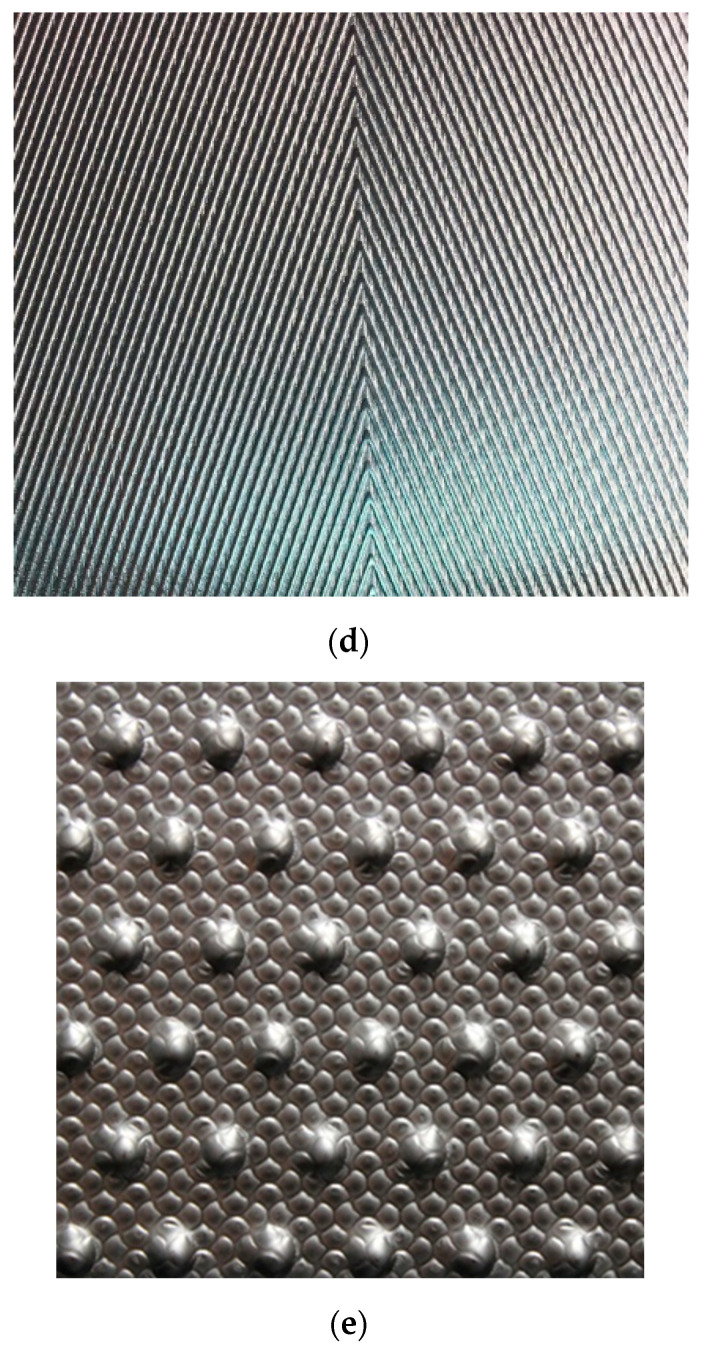
Surface images of the valued tubes: (**a**) EHT-HX; (**b**) EHT-HY; (**c**) EHT-HB/D; (**d**) EHT-HB; (**e**) 1EHT.

Figure 1 presents the experimental setup, and Table 1 summarizes the physical parameters of tubes that were evaluated in this investigation. The first four tubes were used to study the influence of the enhancement structure on heat transfer, while the next three tubes were used to evaluate the effect of tube material on heat transfer, and, finally, the last three tubes were used to evaluate the effect of tube diameter. See [17] for additional experimental results for the first four tube types. See [18] for additional information on the other three tube types. See Figure 2 for an image of the enhancement surface of the evaluated tubes.

A horizontal test section was used with R410a refrigerant flowing on the inside of the tube and water flowing on the outside. Cooling water was measured using a flow meter; it then flowed through the test section and was then returned to the constant temperature water tank. Temperature was measured using a Pt100 platinum resistance temperature sensor. Refrigerant R410a was heated to a predetermined temperature and quality before it entered the test section; it was completely condensed and supercooled in the condenser; flow was measured using the refrigerant flow meter that was located in front of the preheating section and behind the booster pump; Pt100 platinum resistance sensors were installed at the inlet and outlet of preheating and test sections. A pressure gauge in the test section was used to measure the absolute pressure, along with a differential pressure gauge to measure the total differential pressure. There was a 20-channel data acquisition computer system that automatically recorded and stored all measured data.

Experimental conditions include: saturation temperature for condensation was 45 °C, with the mass flux adjusted according to the experimental needs [maximum of 500 kg/(m^2^·s)], an inlet quality controlled at 0.8, and outlet quality maintained at 0.2.

Moffat [19] describes a method to calculate the uncertainty of directly measured and indirectly obtained parameters. Uncertainty in the parameters includes: uncertainty of the diameter was ±0.05 mm; length was ±0.5 mm; current was ±0.1 A; voltage was ±0.1 V; temperature was ±0.1 K; uncertainty of pressure and differential pressure was ±0.075% of the full scale; flow uncertainty was ±0.2% of reading. Calculated parameter relative error of mass flux was ±1.18%; maximum relative error of quality was ±4.13%; and for the HTC, it was ±11.32%.

## 3. Results

### 3.1. Data Analysis

Total heat transfer (see Equation (1)) is obtained from the heat balance of the water outside the tube,

*Q*_t,ts_ = *C*_pl,w,ts_ × *m*_w,ts_ × (*T*_w,ts,in_ − *T*_w,ts,out_) (1)

In Equation (1), *m*_w,ts_ is the water flow in the test section; *c*_pl,w,ts_ is the average specific heat capacity of water; *T*_w,ts,out_ is the outlet temperature of water in the test section; and *T*_w,ts,in_ is the inlet temperature of water in the test section.

Condensation HTC is obtained using Equations (2)–(7). The quality of the refrigerant in the inlet test section, *x*_in_, is calculated from the heat exchange volume of the water in the preheating section, where the total heat transfer (see Equation (2)) of the refrigerant, *Q*_t,ph_, consists of the liquid phase sensible heat (see Equation (3)) of the refrigerant, *Q*_sens_, and the liquid-gas phase transition (see Equation (4)) latent heat, *Q*_lat_.

*Q*_t,ph_ = *C*_pl,w,ph_ × *m*_w,ph_ × (*T*_w,ph,in_ − *T*_w,ph,out_) = *Q*_sens_ + *Q*_lat_ (2)

For Equation (2), *m*_w,ph_ is the water flow in the preheating section; *c*_pl,w,ph_ is the average specific heat capacity of the water; *T*_w,ph,out_ is the outlet temperature of the water in the preheating section; and *T*_w,ph,in_ is the inlet temperature of the water in the preheating section.

*Q*_sens_ = *C*_pl,ref_ × *m*_ref_ × (*T*_sat_ − *T*_ref,ph,in_) (3)

In Equation (3), *m*_ref_ is the refrigerant mass flow rate; *c*_pl,ref_ is the average specific heat capacity of the refrigerant; *T*_sat_ is the refrigerant saturation temperature; and *T*_ref,ph,in_ is the inlet temperature of the refrigerant in the preheating section.

*Q*_lat_ = *m*_ref_ × *h*_lv_ × *x*_in_ (4)

For Equation (4), *h*_lv_ is the latent heat of the refrigerant.

The quality in the outlet test section, *x*_out_, is calculated from Equation (5).

*x*_out_ = *x*_in_ + *Q*_t,ts_/(*m*_ref_ × *h*_lv_) (5)

Inlet and outlet water temperatures and the saturated temperature of the refrigerant inside the tube are factors used to determine the logarithmic mean temperature difference (LMTD) [20,21] (see Equation (6)).

(6) $$LMTD=\frac{\left(T_{w,\mathrm{ts},\mathrm{in}}-T_{\mathrm{sat}}\right)-\left(T_{w,\mathrm{ts},\mathrm{out}}-T_{\mathrm{sat}}\right)}{\ln\left[\left(T_{w,\mathrm{ts},\mathrm{in}}-T_{\mathrm{sat}}\right)/\left(T_{w,\mathrm{ts},\mathrm{out}}-T_{\mathrm{sat}}\right)\right]}$$

Since the selected enhanced tubes being tested are brand new products that have not been used, the fouling thermal resistance can be ignored. Under the condition of ignoring fouling thermal resistance, the HTC of the inside tubes can be calculated using Equation (7).

(7) $$h_{\mathrm{co}}=\frac{1}{A_{\mathrm{ni}}\left(\frac{Q_{t,\mathrm{ts}}}{LMTD}-\frac{1}{A_{0}h_{0}}-\frac{d_{0}\ln\left(\frac{d_{0}}{d_{i}}\right)}{2k_{\mathrm{wall}}A_{0}}\right)}$$

In Equation (7), *A*_ni_ is the actual heat transfer area of the enhanced tubes; *A*_0_ is the outer surface area of the evaluated tube; and *d*_0_ is the outer diameter of the enhanced tube.

Previous research has demonstrated that the Gnielinski correlation [22] can be used to compute the turbulent, single-phase HTC for a smooth tube. Equation (8) presents the Gnielinski correlation that can be used to calculate the HTC of water on the outside of the smooth tube.

(8) $$h_{0}=\frac{\left(f/2\right)\left(Re-1000\right)Pr}{1+12.7{\left(f/2\right)}^{1/2}\left(Pr^{2/3}-1\right)}{\left(\frac{\mu_{\mathrm{bulk}}}{\mu_{w}}\right)}^{0.14}\frac{k_{w}}{d_{h}}$$

Fanning friction (see Equation (9)) coefficient, *f* is calculated using the Petukhov correlation [23] (applicable for smooth tubes in the Re range: 3000 < *Re* < 5 × 10^6^).

*f* = (1.58ln*Re* − 3.28)^−2^ (9)

The surface enhancement of the inner and outer surfaces of the evaluated tube will affect the results. Therefore, the Gnielinski water-side HTC must be modified (using the Wilson graphic method) using the heat transfer enhancement coefficient *C* (the ratio of the water-side HTC of the enhanced tube to the HTC of the smooth tube); the modified Gnielinski formula is given in Equation (10).

(10) $$\frac{1}{Ch_{0}}=\frac{1}{U}-\frac{d_{0}}{d_{i}h_{\mathrm{ev}}}-\frac{d_{0}\ln\left(d_{0}/d_{i}\right)}{2k_{wall}}$$

In Equation (10), *U* is the total HTC.

The friction pressure drop, ∆*P*_f_, is calculated using Equation (11).

∆*P*_f_ = ∆*P*_t_ − ∆*P*_g_ − ∆*P*_m_ − ∆*P*_se_ − ∆*P*_sc_ (11)

In Equation (11), ∆*P*_t_ is the total pressure drop; ∆*P*_g_ is the gravitational pressure drop; ∆*P*_m_ is the dynamic pressure drop; ∆*P*_se_ is the sudden expansion pressure drop; and ∆*P*_sc_ is the sudden contraction pressure drop. In this testing, all the tubes evaluated are placed horizontally, therefore, ∆*P*_g_ is equal to 0.

Equation (12) is used to calculate ∆*P*_m_.

(12) $$\Delta P_{m}=G^{2}\left\{{\left[\frac{x}{\rho_{v}\varepsilon }-\frac{{\left(1-x\right)}^{2}}{\rho_{l}\left(1-\varepsilon \right)}\right]}_{\mathrm{out}}-{\left[\frac{x}{\rho_{v}\varepsilon }-\frac{{\left(1-x\right)}^{2}}{\rho_{l}\left(1-\varepsilon \right)}\right]}_{\mathrm{in}}\right\}$$

In Equation (12), *G* is the mass flux rate; *x* is the quality of the refrigerant; ε is the cavitation rate; *ρ*_v_ is the gas density of the refrigerant; *ρ*_l_ is the density of the refrigerant liquid; ε is calculated by Rouhani et al. [24] and shown in Equation (13).

(13) $$\varepsilon =\frac{x}{\rho_{v}}{\left\{\left[1+0.12\left(1-x\right)\right]\left(\frac{x}{\rho_{v}}+\frac{1-x}{\rho_{l}}\right)+\frac{1.18\left(1-x\right)\left[g\sigma {\left(\rho_{l}-\rho_{v}\right)}^{0.25}\right]}{G\rho_{l}^{0.5}}\right\}}^{-1}$$

Equation (14) can be used to calculate ∆*P*_se_ and Equation (15) is used to calculate ∆*P*_sc_.

(14) $$\Delta P_{\mathrm{sc}}=\frac{G^{2}}{2\rho_{l}}\left[1-\left(\frac{\rho_{l}-\rho_{v}}{\rho_{v}}\right)\right]$$

(15) $$\Delta P_{\mathrm{se}}=\frac{G^{2}\zeta \left(1-\zeta \right)}{\rho_{l}}\left[1-\left(\frac{\rho_{l}-\rho_{v}}{\rho_{v}}\right)\right]$$

In Equation (15), ζ is the area ratio.

### 3.2. Evaluation of Smooth Tube Condensation Correlations

**Figure 3:**
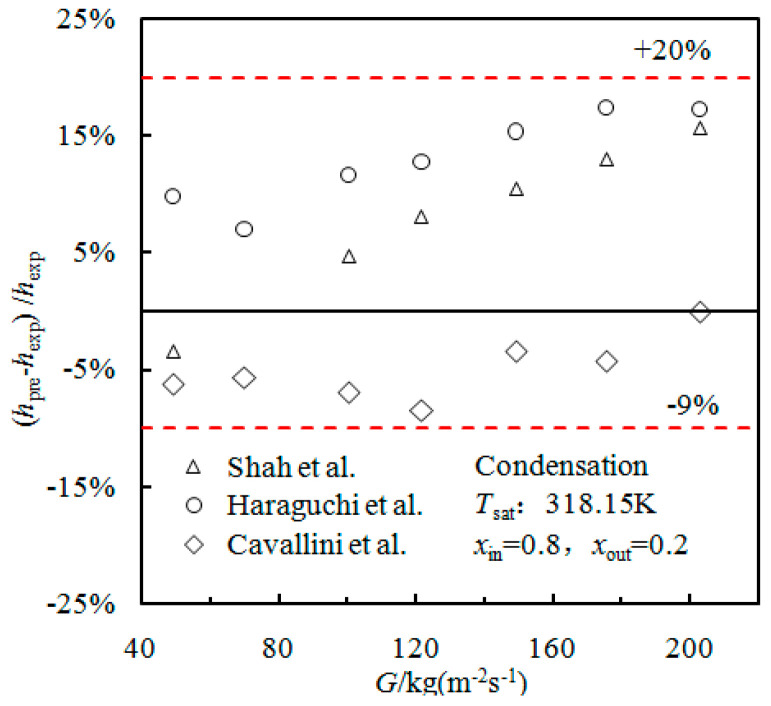
Comparison of experimental data and smooth tube condensation correlations.

Shah et al. [25], Cavallini et al. [26], and Haraguchi et al. [27] present condensation correlations for smooth tubes. In this study, these three smooth tube correlations were compared in Figure 3 with experimental cavitation rate condensation results. The deviation of the HTC was predicted by Cavallini et al. [26] and is within ±9%. The deviation predicted by Shah et al. [25] and Haraguchi et al. [27] is in the range of −9% to +20%.

### 3.3. Condensation Enhanced Heat Transfer Factor in Enhanced Tubes

By introducing the heat transfer enhancement factor (*EF*_h_), the heat transfer of the enhanced and smooth tube is compared (see Equation (16)). This factor is defined as the ratio of HTC of the enhanced tube to the HTC of an equal-diameter smooth tube under the same working conditions.

*EF*_h_ = *h*_e_/*h*_s_ (16)

In Equation (16), *h*_e_ is the HTC of enhanced tubes, and *h*_s_ is the HTC of the smooth tube.

**Figure 4:**
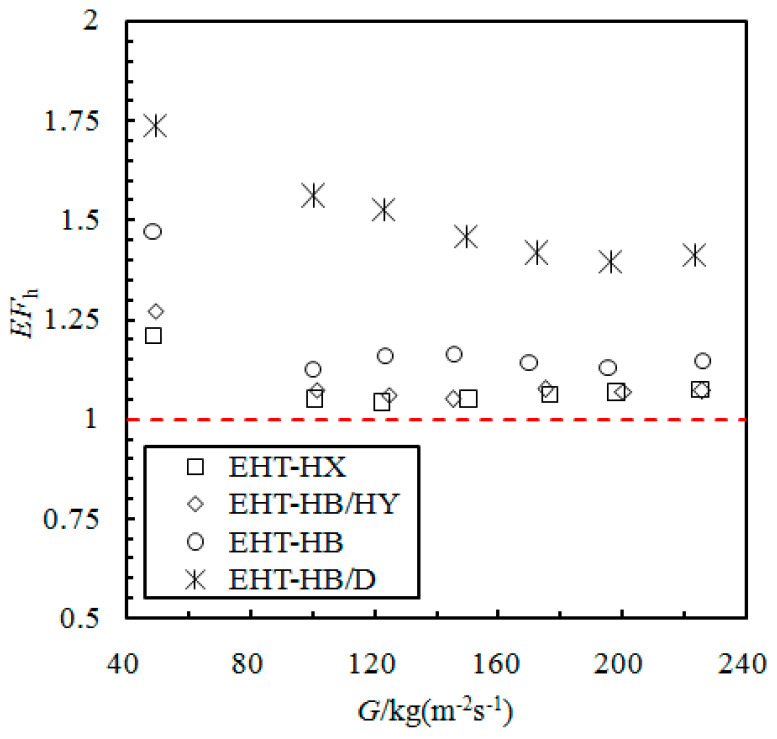
Comparison of enhancement factors of the evaluated enhanced tubes.

Figure 4 compares the *EF*_h_ of the enhanced tubes. The data are from tubes 1 to 4 in Table 1. All the enhancement factors are greater than 1. At low flow rates, the trend of the *EF*_h_ decreases, while for mass flow rates greater than 120 kg/(m^2^·s), the *EF*_h_ trend decreases slowly.

The *EF*_h_ of composite surface-enhanced tubes (EHT-HB/D and EHT-HB/HY) are quite different; the *EF*_h_ of an EHT-HB/D tube ranges from 1.4 to 1.75. This enhancement in the EHT-HB/D tube is the result of the surface producing an increase in the disturbance of the fluid; additionally, it also produces an increase in the turbulence intensity and improves the liquid drainage effect. The *EF*_h_ of the EHT-HB/HY tube can reach 1.27; this is lower than that of the EHT-HB/D tube. This may be a result of the EHT-HB/HY tube fin structure; the HB/HY structure makes it difficult to remove the liquid at the hydrophobic fin and leads to the local heat transfer resistance increasing.

The heat transfer enhancement factors of the non-composite surface enhanced tubes (EHT-HX and EHT-HB) are also different; the *EF*_h_ of the EHT-HB tube is higher than that of the EHT-HX tube. The fin structure in the EHT-HB tube makes it easier for the liquid to flow from the channel groove at the top of the fin; this creates a stronger disturbance in the flow process, thereby improving its HTC.

### 3.4. Evaluation of Enhanced Heat Transfer Performance Factor

The dimensionless parameter, performance factor (*PF*), is the enhanced heat transfer ratio (between enhanced and smooth tubes) divided by the pressure drop ratio. The following formula is used to calculate *PF*:

*PF* = (*h*_e_/*h*_s_) × (*p*_s_/*p*_e_) (17)

In Equation (17), *p*_e_ is the frictional pressure drop of the enhanced tubes, and *p*_s_ is the frictional pressure drop of the smooth tubes under the same working conditions.

**Figure 5:**
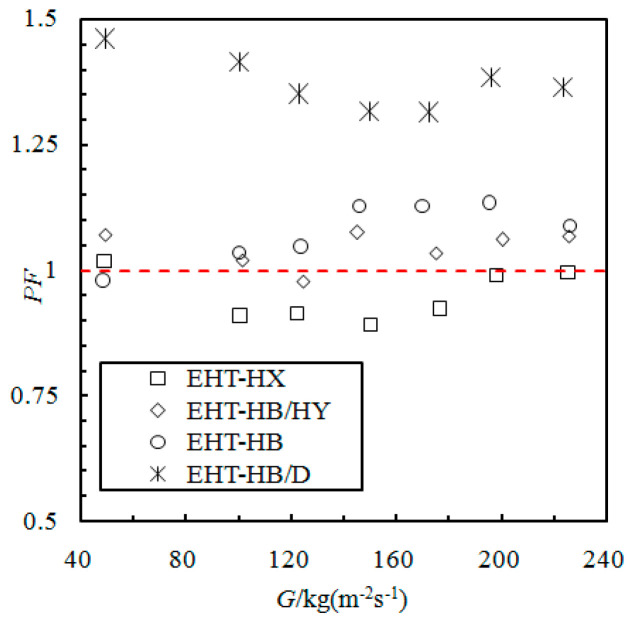
Comparison of performance factors of the evaluated enhanced tubes.

Figure 5 presents the *PF* of the evaluated enhanced tubes. The data are from tubes 1 to 4 in Table 1. The performance factors of the EHT-HB/D and EHT-HB tubes are greater than 1; the *PF* of the EHT-HB/HY tube is slightly larger than 1; and finally, the *PF* of the EHT-HX tube is less than 1. The *PF* of the EHT-HB/D tube is the highest, reaching 1.3~1.5; this indicates that the surface structure can ensure high heat transfer performance and low frictional pressure drop. However, the *PF* of the EHT-HX tube is only 0.9~1.0, and the enhancement of heat transfer is not seen.

With increasing mass flow rate, the *PF* of the EHT-HB/D and EHT-HX tubes decreases initially and then increases, showing a parabolic shape in the low middle and higher values at both ends. The EHT-HB tube shows an initial rise, followed by a decrease; the curve shape is high in the middle and lower at the ends. Finally, the EHT-HB/D tube has the best condensation heat transfer performance, and the heat transfer of the EHT-HB/HY tube is not as good as that of the EHT-HB tube, while the EHT-HX tube has the worst performance factor curve.

### 3.5. Evaluation of Condensation Correlations in Enhanced Tubes

**Figure 6:**
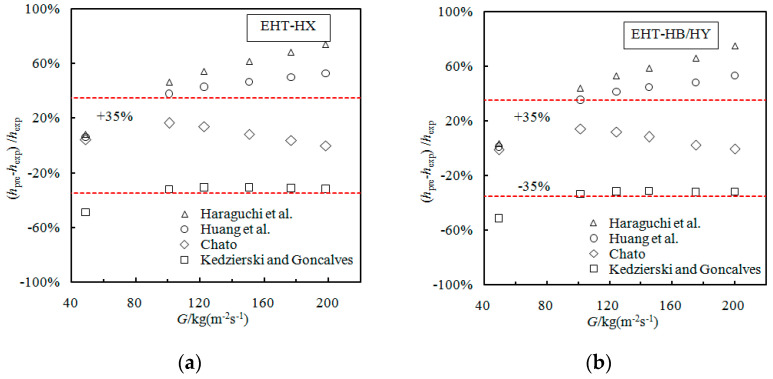

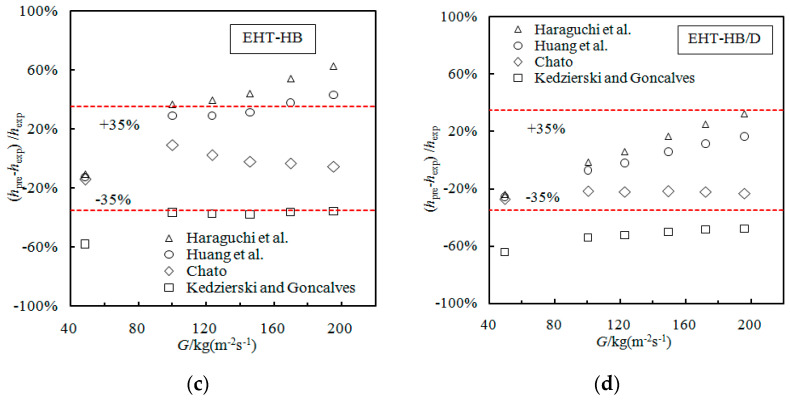
Comparison of using Haraguchi et al. [27]; Huang et al. [28]; Chato [29]; Kedzierski and Goncalves [30] correlations to predict performance for (**a**) EHT-HX, (**b**) EHT-HB/HY, (**c**) EHT-HB, and (**d**) EHT-HB/D.

In previous condensation heat transfer studies, several researchers have proposed condensation heat transfer prediction models. However, the applicability of these models for use with enhanced heat transfer tubes remains to be verified. In this study, four models (Haraguchi et al. [27]; Huang et al. [28]; Chato [29]; Kedzierski and Goncalves [30]) are compared with the enhanced condensation data of this study. Results of that comparison are presented in Figure 6. The data are from tubes 1 to 4 in Table 1.

From Figure 6, it can be seen that the predicted trends of the four models for use in predicting the EHT-HX and EHT-HB/HY tubes are consistent (trends are also consistent with the trends from the previous EF analysis); this is the result of the *EF*_h_ values for the EHT-HX and EHT-HB/HY tubes almost overlapping. Additionally, the *EF*_h_ of the EHT-HB tube is slightly larger than that of the EHT-HX and EHT-HB/HY tubes; therefore, all four models produce the same trend for these types of tubes.

When considering the EHT-HX, EHT-HB/HY, and EHT-HB tubes, the deviation of all of the data points predicted by the Chato [29] model is within ±15%. For the EHT-HB/D tube, the deviation of all data points predicted by the Huang et al. [28] model is within ±30%, while for the Haraguchi et al. [27] model it is within ± 40%.

**Figure 7:**
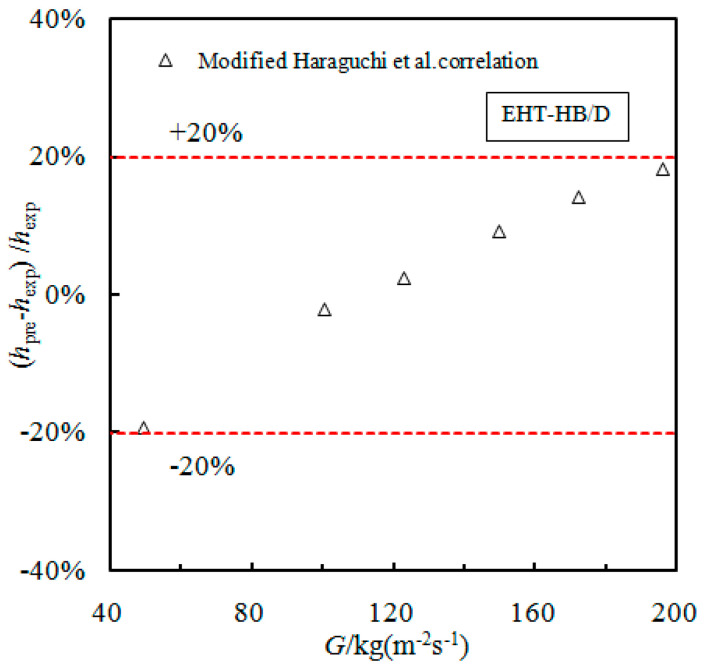
Comparison of the modified Haraguchi et al. correlation for use with the EHT-HB/D tube.

Since the HTC of the EHT-HB/D tube is the largest, the prediction accuracy of existing models is not ideal. It is noted that the Huang et al. [28] model can be seen as a modification of the Haraguchi et al. [27] model (see Table 2), with the Haraguchi et al. model data being based on R22, R134a, R123, etc.; additionally, the mass flow and Reynolds number cover a wide range. Therefore, it is necessary to modify the Haraguchi et al. model in order to make it applicable to the EHT-HB/D tube. The prediction results of the modified Haraguchi et al. model for the HB/D tube can be seen in Figure 7. Here, it can be seen that the modified Haraguchi et al. model can predict that 100% of the data points are within ±20%.

### 3.6. Effect of Tube Material on Enhanced Heat Transfer Performance

**Figure 8:**
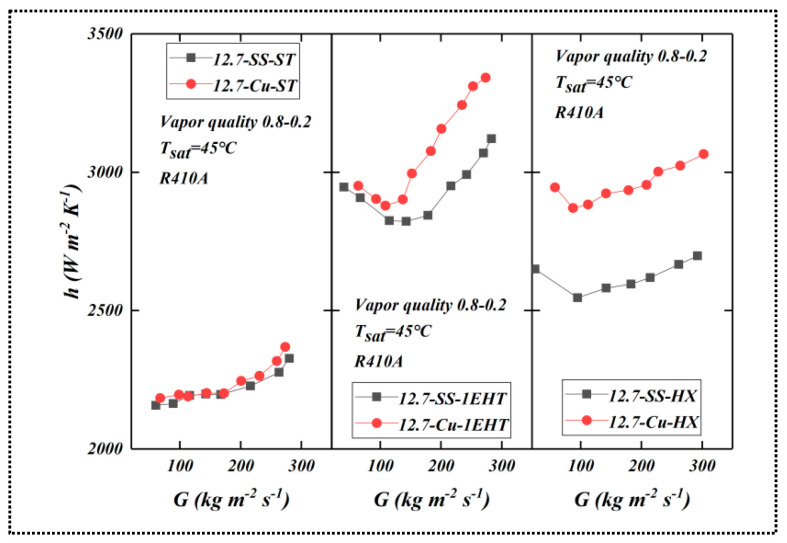
Comparison of HTC (as a function of mass flow rate) for tubes of the same diameter (12.7 mm) produced of different materials for smooth (ST) and enhanced (1EHT and HX) tubes.

Figure 8 compares the HTC as a function of mass flow rate for tubes (of the same diameter) produced from different materials. The data are from tubes 5 to 7 in Table 1. For smooth tubes, the HTC values of copper tubes and SS are similar (the copper tube HTC values are slightly higher). For enhanced tubes, the performance trends are different; the HTC of the copper tube is larger than the SS tube.

Thermal conductivity varies between copper and SS; this affects the temperature distribution in the enhanced surface (fins); the higher the thermal conductivity of the material, the closer the temperature of the fin is to the temperature of the fin root. For low-conductivity materials, the opposite is true: the temperature of the fin deviates more from the temperature of the fin root. The temperature difference at different positions of the fins affects the efficiency. Fin efficiency is positively correlated with the thermal conductivity of the material, thus affecting the HTC. Therefore, the HTC of copper tube is higher than that of SS tube. It can also be seen from the figure that the influence of tube material composition on the HTC of the EHT-HX tube is larger; the tube produced from copper is significantly higher than SS tubes. Differences in performance between enhancement types are also noted; however, this is a function of differences in the area enhancement ratio between the EHT-HX and 1EHT tubes; additionally, there are differences in the enhancement characters used. The former has a higher area ratio than the latter; therefore, temperature difference and fin efficiency have a greater effect on performance.

For smooth tubes, the HTC increases with the increase in mass flow rate, and in the middle region, the increase is relatively slow. However, for the enhanced tube, the HTC initially decreases and then increases; they reach their minimum values near 100 kg/(m^2^·s). This phenomenon is related to flow pattern, shear force, surface tension, etc., and requires further research.

**Figure 9:**
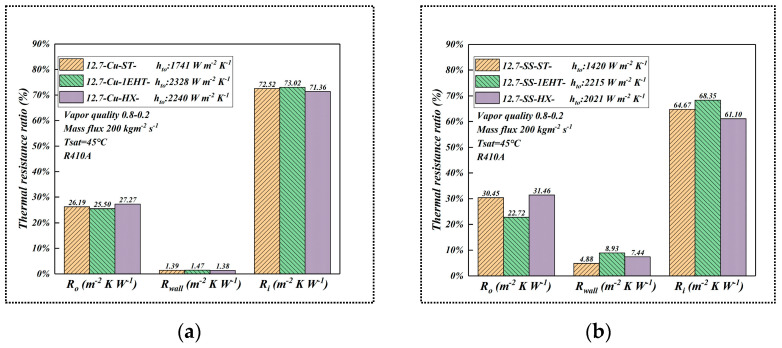
Comparison of condensation thermal resistance ratio of different tube materials (**a**) copper (**b**) SS.

Figure 9 shows the comparison of heat transfer resistance values (copper and SS) that take place during condensation when using enhanced tubes. The data are from tubes 5 to 7 in Table 1. From Figure 9, it can be concluded that the thermal resistance of the tube material is the least important component when looking at the total thermal resistance of the tube; however, the total thermal resistance is still an important component. The thermal resistance ratio of SS tube is higher than that of copper tube; additionally, the 1EHT tube is enhanced on both sides (inside and outside), therefore its external heat resistance is lower than that of either the smooth tube or the EHT-HX tube.

**Figure 10:**
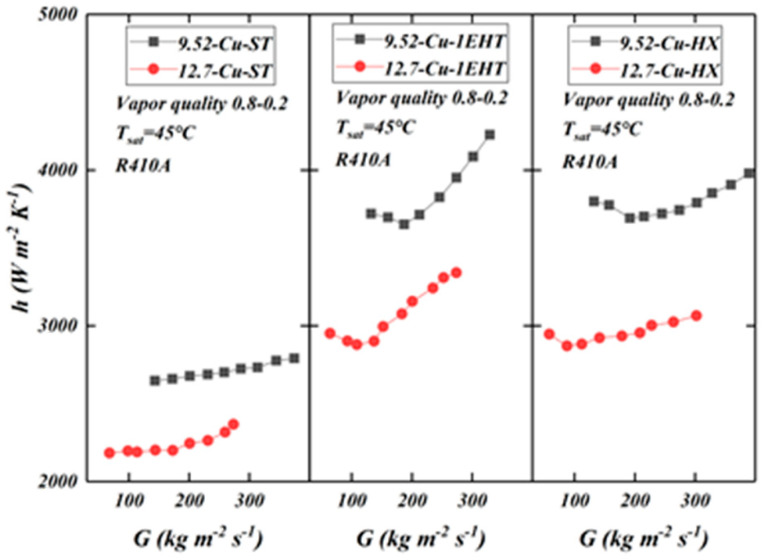
Comparison of HTC (as a function of mass flow rate) for different diameter (9.52 and 12.7 mm) tubes produced from copper, for smooth (ST) and enhanced (1EHT and HX) tubes.

According to Figure 10, it can be seen that the tube diameter and material will affect the HTC. The data are from tubes 5 to 7 in Table 1. Larger values of the HTC in the 9.52 mm tube are seen when compared with the HTC values in the 12.7 mm tube. The HTCs of the enhanced tubes initially decrease and then increase; however, the turning point for the small tube diameter is delayed. Gravity is playing a leading role in heat transfer that takes place in the tube with the larger diameter, while in the smaller tube diameter, shear force and surface tension play the leading roles; this is conducive to the uniform distribution of the liquid film on the tube diameter. Additionally, in the smaller tube diameter, there is a larger heat flux per unit volume; all these factors contribute to the larger HTC that is shown in the enhanced 9.52 mm tube (when compared with the HTC of the 12.7 mm tube).

### 3.7. Influence of Tube Diameter on Condensation HTC

**Figure 11:**
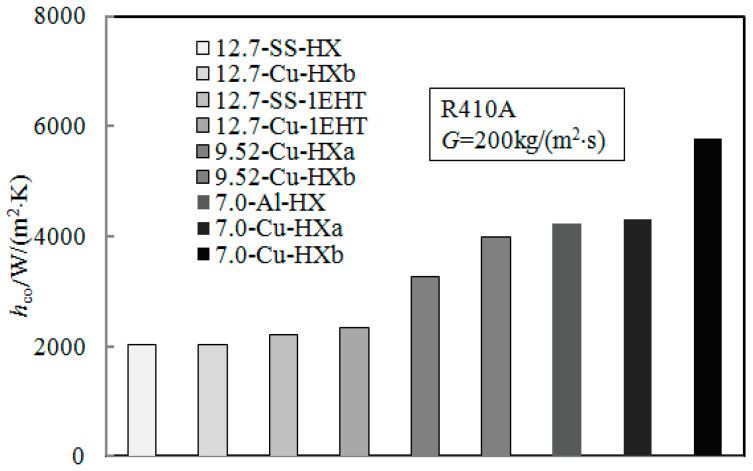
Effect of tube diameter on condensation HTC.

Figure 11 shows the effect of tube diameter on condensation HTC. The data are from tubes 5 to 10 in Table 1. It can be seen that with the decrease in tube diameter, the condensation HTC in the enhanced tube shows an increasing trend; additionally, the tube diameter has a greater impact on the condensation HTC than the enhanced surface structure. This can be explained as follows: with the decrease in tube diameter, shear force and surface tension gradually replace gravity and become the dominant forces. This is beneficial to remove and dilute the liquid film at the bottom. The smaller the tube diameter, the higher the surface area density (surface area volume ratio); this results in a higher heat flux per unit volume.

## 4. Conclusions

Through experimental methods, the condensation of R410A in different enhanced tubes was studied, and HTC and pressure drop data were obtained. Through a series of analyses, the following conclusions were obtained:

(1) The *EF*_h_ of the EHT-HB/D tube is the highest; its performance is closely related to increasing fluid disturbance and improving drainage. The structure of the EHT-HB/HY tube increases the local thermal resistance and inhibits heat transfer, while the drainage effect of the EHT-HB tube is better than that of the EHT-HX tube.
(2) The best overall condensation heat transfer resistance characteristics (and the highest PF) are shown in the EHT-HB/D tube; it has a low friction pressure drop and high heat transfer performance. Additionally, the *PF* of the EHT-HB tube is greater than one, the *PF* of EHT-HB/HY is slightly higher than one, and the *PF* of EHT-HX is less than one. In general, when the mass flow rate increases gradually, the *PF* initially decreases and then increases.
(3) Correlations that predict the condensation HTC of enhanced tubes are discussed and modified. For EHT-HX, EHT-HB/HY, and EHT-HB tubes, the deviation of all data points predicted by the Chato model is within ±15%. However, for the EHT-HB/D tube, a modification to the Haraguchi et al. model is necessary, and that modification can predict that 100% of the data points are within ±20%.
(4) Thermal conductivity (SS and copper) of the tube material for smooth tubes has a minimal effect on its thermal hydraulic performance; however, for the enhanced tubes, the HTC increases with increasing thermal conductivity in extreme environments of condensation flow, at the saturation temperature of 318.15K with a saturation pressure of 2733.5 kPa. The refrigerant side convective heat transfer resistance dominates, while the wall heat transfer resistance makes up a modest fraction of the overall resistance. The thermal conductivity of the enhancement character (fin) affects the heat transfer for enhanced tubes.
(5) The effect of tube diameter on condensation heat transfer in the enhanced tubes with different tube diameters is much higher than that of the enhancement surface structure. With the reduction of tube diameter, shear force and surface tension gradually replace gravity and become the dominant forces; this is conducive to the removal of the liquid film at the bottom.

Future research will continue this study and also provide a simulation of this work.

## Figures and Tables

**Table 1:** Physical parameters of tubes evaluated in this study.

Parameters	EHT-HX	EHT-HB	EHT-HB/D	EHT-HB/HY	Smooth	1EHT	EHT-HX	EHT-HX	EHT-HX	EHT-HX
Material	SS	SS	SS	SS	Cu/SS	Cu/SS	Cu/SS	Al	Cu	Cu
Outer diameter (mm)	12.7	12.7	12.7	12.7	9.52/12.7	9.52/12.7	9.52/12.7	7.0	7.0	7.0
Thickness (mm)	0.6	0.6	0.6	0.6	0.61	0.61	0.61	0.47	0.22	0.24
Length (mm)	2	2	2	2	2	2	2	2	2	2
Dimple/fin height, mm	1.14	0.08	1.21	0.08	-	0.19/1.71	0.25	0.06	0.10	0.15
Dimple/fin pitch, mm	5	0.8	4	0.8	-	0.35/1.34	0.31	0.32	0.32	0.35
Dimple/fin width, mm	2.3	0.31	3.51	0.31	-	4	0.8	0.07	0.06	0.17
Helix angle, °	26.2	21	63	21	-	60	21	0	16	30

**Table 2:** Modified Haraguchi et al. correlation.

Author	Correlation
Modified Haraguchi et al. correlation	$h={\left(h_{F}^{2}+h_{B}^{2}\right)}^{\frac{1}{2}}$ $h_{F}=0.0152\left(1+0.6Pr_{l}^{0.8}\right)\left(\frac{\Phi_{V}}{X_{\mathrm{tt}}}\right)Re_{l}^{0.77}\frac{k_{l}}{d_{h}}$ $h_{B}=0.790H\left(\varepsilon \right){\left(\frac{GaPr_{l}}{Ja_{l}}\right)}^{\frac{1}{4}}\frac{k_{l}}{d_{h}}$ $\Phi_{V}=1+0.5{\left[\frac{G}{\sqrt{gd_{h}\rho_{g}\left(\rho_{l}-\rho_{g}\right)}}\right]}^{0.75}X_{\mathrm{tt}}^{0.35}$ $Ga=\frac{8\rho_{l}\left(\rho_{l}-\rho_{g}\right)d_{h}^{3}}{\mu_{l}^{2}}$ $H\left(\varepsilon \right)=\varepsilon +\left\{10\left[{\left(1-\varepsilon \right)}^{0.1}-1\right]+1.7\times 10^{-4}Re_{lo}\right\}\sqrt{\varepsilon }\left(1-\sqrt{\varepsilon }\right)$ $\varepsilon ={\left[1+\frac{\rho_{g}}{\rho_{l}}\left(\frac{1-x}{x}\right)\left(0.4+0.6\sqrt{\frac{\frac{\rho_{l}}{\rho_{g}}+0.4\left(\frac{1}{x}-1\right)}{1+0.4\left(\frac{1}{x}-1\right)}}\right)\right]}^{-1}$ Applicable to the EHT-HB/D tube

## Data Availability

The pre-processed data used in this study are available on request from the corresponding author.

## Nomenclature

*A*	test tube surface area, m^2^
*C*	enhancement ratio
*c_p_*	specific heat, J/(kg·K)
*D*	dimple
*d*	test tube diameter, m
*d_h_*	hydraulic diameter, m
*Fa*	(*ρ*_l_−*ρ*_v_) *σ*/*G*^2^*D*_h_
*f*	Fanning friction factor
*G*	mass flux, kg/(m^2^·s)
*Ga*	Galileo number
*g*	gravitational acceleration, m/s^2^
HB	herringbone
HB/D	herringbone dimple
HB/HY	hydrophobic herringbone
HX	spiral microgrooves
*h*	heat transfer coefficient, W/(m^2^·K)
*h_lv_*	latent heat of vaporization, J/kg
*k*	thermal conductivity, W/(m·K)
*Ja*	Jakob number
*l*	liquid only
*L*	tube length, m
*LMTD*	logarithmic mean temperature, K
*m*	mass flux, kg/s
*M*	molecular weight
*P*	Pressure, kPa
*PF*	performance factor
*Pr*	Prandtl number
*Q*	heat transfer amount, W
*q*	heat flux, W/m^2^
*Re*	Reynolds number
*T*/*t*	temperature, K/°C
*U*	Total heat transfer coefficient, W/(m^2^·K)
*x*	vapor quality
*X_tt_*	Martinelli parameter $X_{\mathrm{tt}}={\left(\frac{1-x}{x}\right)}^{0.9}{\left(\frac{\rho_{v}}{\rho_{l}}\right)}^{0.5}{\left(\frac{\mu_{l}}{\mu_{v}}\right)}^{0.1}$
*Greek symbols*	
*μ*	dynamic viscosity, Pa·s
*ρ*	density, kg/m^3^
*ε*	void fraction
*σ*	surface tension, N/m
*ζ*	area ratio
*Φ*	two-phase multiplier
*Subscripts*	
*bulk*	bulk temperature
*exp*	experimental
*f*	frictional
*g*	gravitational
*i*	inner
*in*	inlet
*l*	liquid phase
*lat*	latent heat
*m*	momentum
*ni*	actual heat transfer area
*o*	outer
*out*	outlet
*ph*	preheating section
*pre*	predictive
*ref*	refrigerant
*s*	smooth
*sat*	saturated
*sc*	sudden contraction
*se*	sudden enlargement
*sens*	sensible heat
*t*	total
*te*	test section
*tp*	two-phase
*ts*	test section
*v*	vapor phase
*wall*	wall parameters
*w*	water
